# Supplementary material for: Scaling crossover in droplet impact force on elastic substrates
Source: Nat Commun. 2026 Jan 28;17:607. doi: 10.1038/s41467-025-67790-6 (PMC12852737; doi:10.1038/s41467-025-67790-6)
Supplement: Supplementary file 1 — Supplementary Information [file 41467_2025_67790_MOESM1_ESM.pdf]

Supplementary Information

Scaling Crossover in Droplet Impact Force on Elastic Substrates

Yuto Yokoyama[1,2], Hirokazu Maruoka[3], Kaie Matsunuma[2], Yoshiyuki Tagawa[2,4*]

[1]Micro/Bio/Nanofluidics Unit, Okinawa Institute of Science and Technology Graduate University, 1919-1 Tancha, Onna, 9040497, Okinawa, Japan.
[2]Department of Mechanical Systems Engineering, Tokyo University of Agriculture and Technology, 2-24-16 Nakacho, Koganei, 1848588, Tokyo, Japan.
[3]Nonlinear and Non-equilibrium Physics Unit, Okinawa Institute of Science and Technology Graduate University, 1919-1 Tancha, Onna, 9040497, Okinawa, Japan.
[4]Institute of Global Innovation Research, Tokyo University of Agriculture and Technology, 2-24-16 Nakacho, Koganei, 1848588, Tokyo, Japan.

*Corresponding author(s). E-mail(s): tagawayo@cc.tuat.ac.jp;
Contributing authors: yuto.yokoyama@oist.jp; hirokazu.maruoka@oist.jp; s250937s@st.go.tuat.ac.jp;

1 Physical properties of the elastic substrates

The elastic moduli E of the substrates were measured using the indentation method [1, 2]. Specifically, a solid sphere with a radius R (a styrene ball with $R = 7.35$ mm for the polyurethane gels and a steel ball with $R = 3.95$ mm for the gelatin gel) attached to a micrometer gauge was pressed against the substrate, and a load was applied. Using the feed of the gauge, an arbitrary indentation length δ was applied, and the apparent force F was measured using an electronic balance placed beneath

the substrate. E was calculated using Hertzian contact theory [3],

$$E = \frac{3F(1-\nu^2)}{4R^{1/2}\delta^{3/2}}, \tag{1}$$

where ν is the Poisson's ratio of the gel. Here, the deformation of the sphere was neglected, and the Poisson's ratio of the gel was assumed to be $\nu = 0.5$ based on the previous study [4]. δ was changed from approximately 0.1 to 5.0, and the measured F for different substrates are shown in Fig. 1**a**. E is calculated using Eq. 1 from F and is shown in Fig. 1**b**. The dashed lines in Fig. 1**b** indicate the averaged values of E for each substrate, and the average E is used in this study (Table 1 of the main text).

The stress-optic coefficient C is estimated using the procedure developed in our previous study [5]. At the same time as measuring the substrate elasticity described above, the retardation fields within the elastic substrates, according to different loading forces F were measured using the polarizing camera. Under the same conditions as the experiment (E, F, R), the stress field within the substrate can be calculated using Hertzian contact theory [3, 6]. Using the integrated photoelasticity [5], the internal stress field within the substrate is integrated and the retardation field can be calculated with arbitrary stress-optic coefficient C. C is determined so that the error between the calculated retardation field and that obtained in the experiment is minimized. The detailed procedure is described in Ref. [5]. The obtained C for each substrate are shown in Fig. 1, and the dashed lines are the average values of C, which are used in this study (Table 1 of the main text).

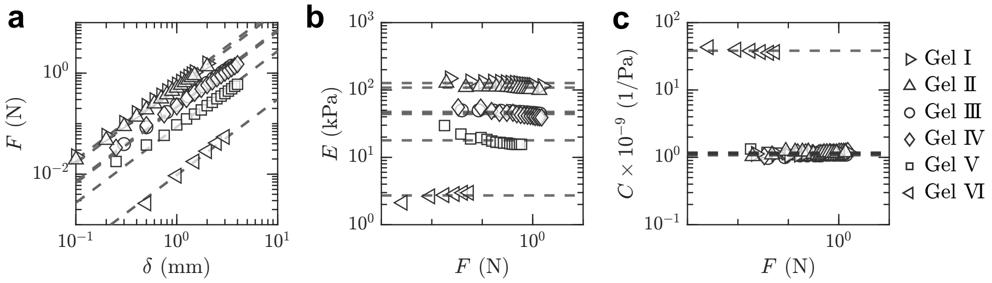

Supplementary Figure 1 Physical properties of the elastic substrates a Loading force F acting on the elastic substrate measured by the electric balance against the surface indentation δ. The dashed lines are calculated by Eq. (1) using the averaged elastic modulus. **b** E against F for different substrates. The dashed lines indicate the average value of each. **c** The stress-optic coefficient C with different loading forces F. The dashed lines indicate the average value of each.

2 Dynamic behaviors of the rigid sphere and the substrate response

Figure 2 shows the stress field in the elastic substrate Gel Ⅲ ($E = 47.4\,\mathrm{kPa}$) induced by the rigid sphere's impact. At $t \simeq 2.0\,\mathrm{ms}$, the negative stress is significant due to the upward motion of the sphere.

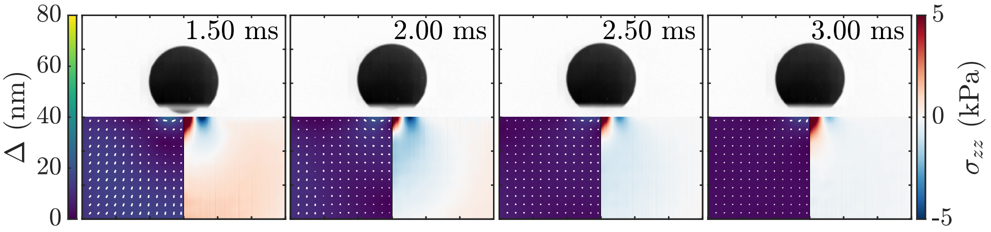

Supplementary Figure 2 Dynamic behaviors of the rigid sphere and the substrate response. Spatio-temporal distribution of the stress field (bottom-left panels) photoelastic parameters, Δ and ϕ, and (bottom-right panels) reconstructed axial stress σ_{zz} when a rigid sphere impacts on the elastic substrate, Gel I ($E = 47.4\,\mathrm{kPa}$) with $V \simeq 2.8 \pm 0.1$ m/s. In the bottom-left panels, the colormap indicates the retardation Δ and white arrows indicate the orientation ϕ. The length of white arrows correspond to the magnitude of retardation shown in the colorbar and corresponds to the one in Fig 1 of the main text. The gray-scale images of the impactor on the upper panel are obtained by subtracting the background image without the impactor.

3 Stress and force acting on the elastic substrate

Figure 3 shows the temporal evolution of the axial stress distribution σ_{zz} on the elastic substrate, Gel Ⅲ ($E = 47.4\,\mathrm{kPa}$) at $z = 0$, generated by the impact of a silicone oil droplet with different viscosities.

4 Stress distribution at $z = 0$

Philippi et al. [7] proposed that the axial stress field acting on the substrate surface, when an inviscid droplet impacts a rigid substrate, is described as

$$\frac{\sigma_{zz}(z=0)}{\rho V^2} = \frac{3}{\pi\sqrt{3r/R - (tV/R)^2}}. \tag{2}$$

The spatio-temporal distribution of the stress field calculated by this equation is shown in Fig. 4 and compared with the experimental data of 10^0 cSt silicone oil droplets impacting onto different substrates. The theoretical distribution exhibits a non-central peak in the axial stress, whereas the experimental data do not. Note that the theory is applicable only to the early stage of the impact Philippi et al. [7]. Cheng et al. [8] demonstrated that the theoretical impact force obtained by integrating Eq.

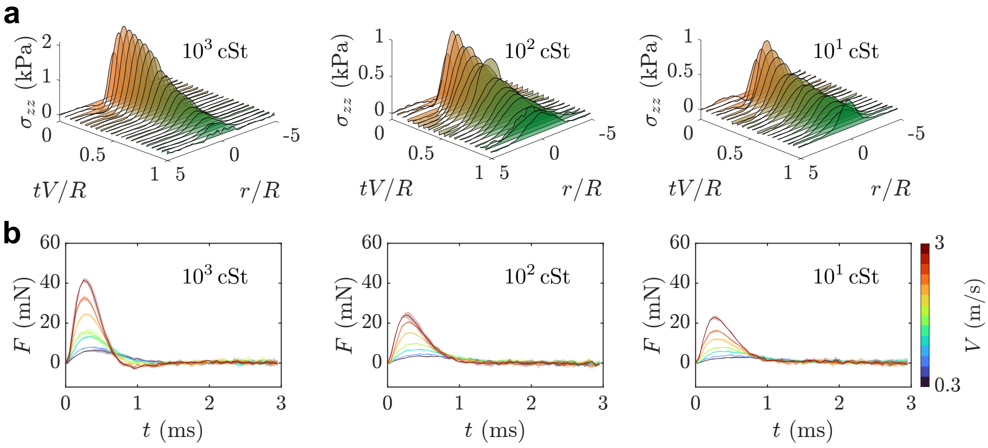

Supplementary Figure 3 Stress and force acting on the elastic substrate. a Temporal evolution of the axial stress distribution σ_{zz} on the elastic substrate, Gel III ($E = 47.4\,\text{kPa}$) at $z = 0$, when Cauchy number, $\text{Ca} = \rho V^2/E$, is approximately 0.02. The data were smoothed by Matlab function "`smoothdata`". **b** Temporal evolution of the impact force with different impact velocities during droplet impact on the elastic substrate, Gel III ($E = 47.4\,\text{kPa}$). The shaded regions represent one standard deviation for three experiments.

(2) follows the experimental force-time curve at the early stage before the maximum impact force occurs, i.e., $tV/R \lesssim 0.34$ for the low-viscosity droplet.

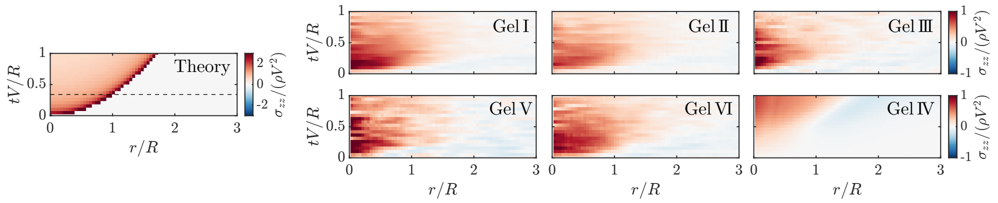

Supplementary Figure 4 Stress distribution at $z = 0$. Comparison between the theoretical and the measured stress fields. The theoretical stress distribution is calculated using Eq. (2) proposed by Philippi et al. [7]. The experimental data are taken at $V \simeq 1.15$ m/s for 10^0 cSt silicone oil droplets. Dashed line indicates $tV/R = 0.34$, which is the typical time when the maximum impact force occurs for a low-viscosity droplet impacting on a rigid substrate [8, 9].

5 Stress and force acting on the softest substrate (Gel VI)

Figure 5**a** shows the stress field in the elastic substrate Gel VI ($E = 2.7$ kPa) induced by the 1 cSt silicone oil droplet's impact. Figure 5**b** shows the temporal evolutions of the impact force induced by the impact of a 1 cSt silicone oil droplet on Gel VI ($E = 2.7$ kPa) with different impact velocities.

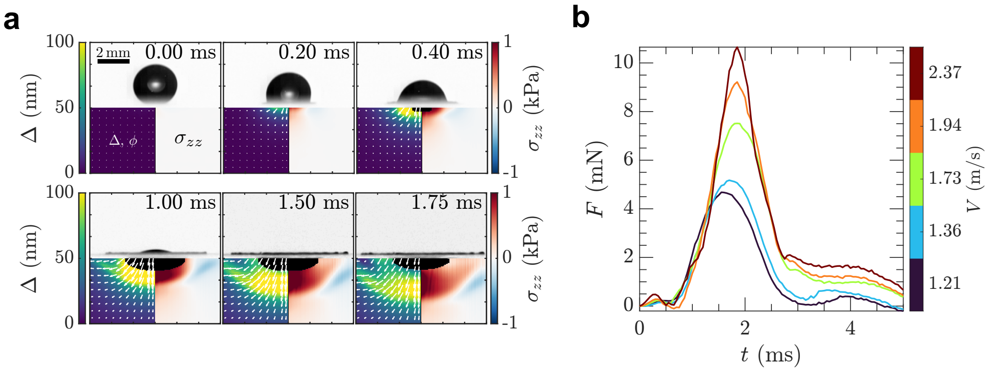

Supplementary Figure 5 Representative reconstructed stress fields and force profile. a Spatio-temporal distribution of the stress field (bottom-left panels) photoelastic parameters, Δ and ϕ, and (bottom-right panels) reconstructed axial stress σ_{zz} when a droplet impact on the elastic substrate Gel VI ($E = 2.7\,\text{kPa}$) with $V \simeq 2.37$ m/s. In the bottom-left panels, the colormap indicates the retardation Δ and white arrows indicate the orientation ϕ. The arrow length corresponds to the magnitude of retardation shown in the colorbars. The gray-scale images of the impactor on the upper panel are obtained by subtracting the background image without the impactor. **b** Temporal evolution of the impact force with different impact velocities during a 1 cSt silicone oil droplet impact on the elastic substrate Gel VI ($E = 2.7\,\text{kPa}$).

6 Parameter space

Figure 6 shows the experimental conditions explored in this study on the Ohnesorge number ($\text{Oh} = \eta/\sqrt{\rho R \gamma}$) and Weber ($\text{We} = \rho V^2 R/\gamma$) plane. It indicates that all the experimental data were taken in the range We > 50, where the second force peak is hard to be pronounced as mentioned in the main text.

7 Effect of water-hammer pressure

The order of magnitude of the force generated by water-hammer pressure can be estimated as [8, 10–12]

$$F_w = \rho R^2 V^3 / c. \tag{3}$$

The ratio of the maximum impact force F_{max} measured in this study to F_w is plotted in the figure below (here, the sound speed in silicone oil is set to $c = 1000$ m/s). We examined the ratio of the maximum impact force F_{max} measured in this study to F_w (see Fig. 7) and confirmed that F_{max}/F_w is much larger than 1 in our experiment. Even in the case where F_{max}/F_w is smallest, it is still approximately 600. it is clear that F_{max} is much larger than F_w in this study. To achieve F_{max}/F_w of approximately 1, a collision velocity of around 100 m/s is required for a typical millimeter-sized droplet impact.

8 Effect of wetting ridge

When a droplet lands on a soft elastic substrate, the substrate near the contact line is lifted vertically by the surface tension of the droplet, resulting in the formation of

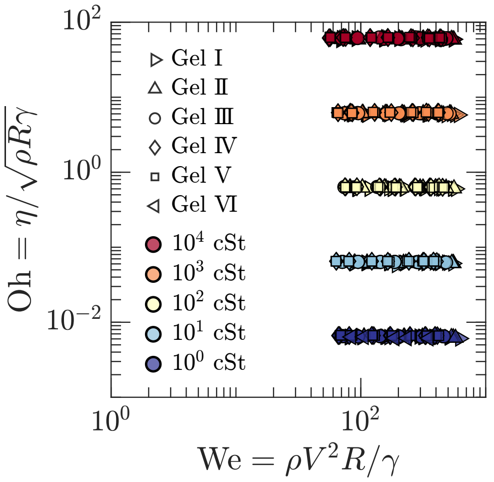

Supplementary Figure 6 Experimental parameter space explored in this study. Experimental conditions explored in this study plotted on the Oh–We plane. The axes are $\mathrm{Oh} = \eta/\sqrt{\rho R \gamma}$ and $\mathrm{We} = \rho V^2 R/\gamma$.

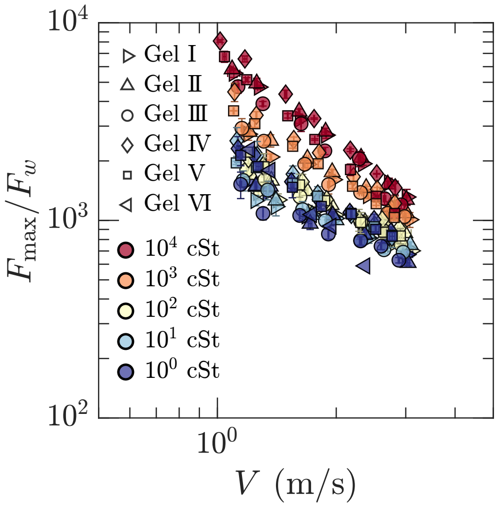

Supplementary Figure 7 Effect of water-hammer pressure Comparison between maximum impact force F_{max} measured in this study and the force generated by water-hammer pressure via Eq. (3)

a wetting ridge [13]. The expected height of the wetting ridge on our softest substrate ($E = 17.9$ kPa) is approximately 1.2 μm, estimated from the elasto-capillary length $l_e = \gamma/E$ [14–16] where γ is the surface tension of the droplet which is typically 20 mN/m for silicone oil droplets. The height is much smaller than the spatial resolution of the current optical setup and therefore not resolved in the images.

The force exerted on the substrate by the wetting ridge can be estimated from the surface tension of the liquid and the length of the contact line. For a silicone oil droplet with a radius of 1 mm, this force is estimated to be approximately 0.13 mN (when the contact angle is 90 degrees). This is more than 10 times smaller than the lowest F_{max} (2 mN) measured in this study. Additionally, the changes in the stress field near the contact line caused by the formation of the wetting ridge are expected to affect an area approximately equal to the elasto-capillary length due to its geometric shape. That is, the stress field changes are considered to disappear in an area approximately 1.2 μm away from the contact line [14]. However, in dynamic wetting such as droplet impact, deformation of the substrate caused by the formation of the wetting ridge has been reported to induce additional energy loss [14]. As a result, this is suggested to dissipate some of the droplet's kinetic energy, thereby influencing the retraction velocity of the droplet after maximum spreading [17, 18]. This may also reduce the impact force or stress acting on the substrate. There is a lack of clear evaluative data to suggest a quantitative discussion at present. Furthermore, given the small magnitude of the force generated by the wetting ridge, as shown above, and the short elasto-capillary length, the contribution to impact force and stress may be secondary within the experimental range of this study.

9 Surface roughness of the acrylic container

To evaluate the possibility of air cushioning in our experimental regime, we conducted AFM measurements on the surface of the acrylic container used as the mold for gel preparation. While the soft gel surface could not be stably scanned due to deformation under the AFM tip, the acrylic surface exhibited a characteristic roughness in the range of 10–100 nm (Fig. 8).

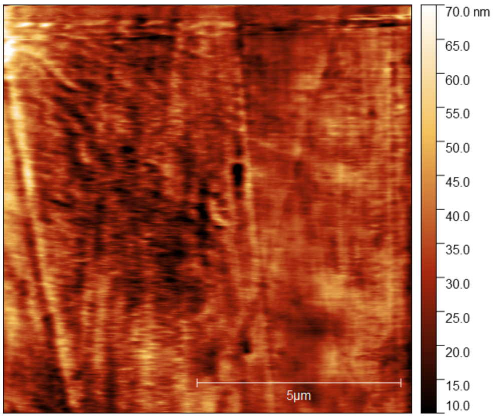

Supplementary Figure 8 Surface characterization of the acrylic mold. Atomic force microscopy (AFM) measurement of the surface of the acrylic container used as the mold for gel preparation.

10 Scaling behavior of maximum impact force

The relationships between Π and Ca for each combination of substrate elasticity and droplet viscosity are best fitted by a power-law model $\Pi = B\mathrm{Ca}^{\beta}$. The best-fit prefactor B and index β are shown in Fig. 9 as a function of θ. The prefactor B seems not to depend on θ, while the index β changes from 1 to 3/5 with increasing θ. Note that, however, a 1 cSt silicone oil droplet impacting on Gel VI ($E = 2.7$ kPa) indicates $\beta \simeq 0.5$, which is close to the index of Hertzian impact scaling, $\beta = 3/5$, although it is a low-viscosity droplet.

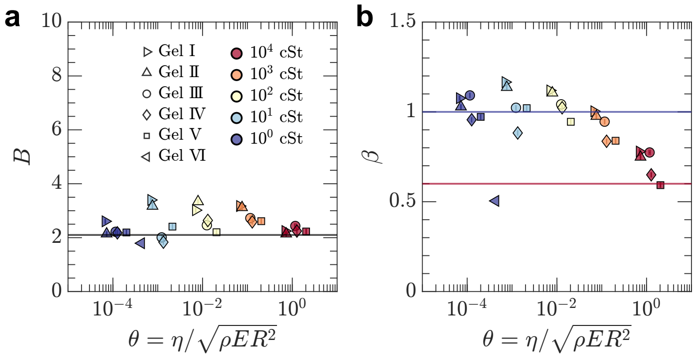

Supplementary Figure 9 Scaling behavior of maximum impact force a Best-fit prefactor B of scaling law $\Pi = B\mathrm{Ca}^{\beta}$ for combinations of substrate elasticity and droplet viscosity. The solid line is a guide to the eye. **b** Best-fit index β of scaling law $\Pi = B\mathrm{Ca}^{\beta}$ for combinations of substrate elasticity and droplet viscosity. The red solid line indicates the index of Hertzian impact scaling, 3/5, and the blue solid line indicates the index of inertial force scaling, 1.

11 Photoelastic tomography

Here, we introduce the stress-reconstruction algorithm based on photoelastic tomography, following our previous work [4]. To reconstruct the stress field (here we consider the axial stress σ_{zz}), we use the photoelastic parameters (the phase retardation $\Delta(x, z)$ and the principal-axis orientation $\phi(x, z)$) measured in two sections parallel to the x–y plane (lower and upper), separated by a distance Δz along the z-axis [19, 20]. Under the small-retardation conditions ($\Delta < \lambda/4$ and rotation of the principal stress direction $< \pi/6$), the stress–optic law gives the following line-integrated relations [21, 22]:

$$V_1 \equiv \Delta \cos 2\phi = C \int_{-\infty}^{\infty} (\sigma_{xx} - \sigma_{zz})\, dy, \tag{4}$$

$$V_2 \equiv \Delta \sin 2\phi = 2C \int_{-\infty}^{\infty} \sigma_{xz}\, dy, \tag{5}$$

where C is the stress–optic coefficient and y is the optical axis of the camera. We denote the quantities measured in the lower and upper planes by unprimed and primed symbols, respectively, i.e. (V_1, V_2) and (V_1', V_2').

Following the above integrated photoelasticity formulation, force equilibrium along the x-direction yields the relation

$$\int_A^B \sigma_{zz}\, dy = \frac{1}{2C\Delta z}\left(\int_x^X V_2'\, dx - \int_x^X V_2\, dx\right) - \frac{1}{C}V_1, \tag{6}$$

where A and B denote the boundary of the stressed volume on the y-axis, and X is the outermost position where the stress vanishes. Equation (6) expresses the integral of σ_{zz} along y in terms of the measured photoelastic parameters.

We now assume an axisymmetric stress field about the z-axis (normal impact onto a flat substrate), so that all stress components depend only on the radial coordinate r and z. For a given z and time t, we discretize the x-direction into N rays with spacing Δx, and consider N concentric rings of thickness $\Delta r = \Delta x$ with radii

$$r_i = i\,\Delta r, \qquad i = 1, \ldots, N. \tag{7}$$

At each radial position r_i we define the discrete quantities $V_1^{(i)}$, $V_2^{(i)}$, and $V_2'^{(i)}$, obtained directly from the photoelastic measurement.

The discrete counterpart of Eq. (6) at radius r_i is then written as

$$V_1^{(i)} - \frac{\Delta x}{2\Delta z}\sum_{j=1}^{i}\left(V_2'^{(j)} - V_2^{(j)}\right) = 2C\sum_{j=1}^{i} W_{i,j}\,\sigma_{zz}^{(j)}, \tag{8}$$

where $\sigma_{zz}^{(j)}$ is the axial stress in the jth ring and $W_{i,j}$ is the path length of the ray with impact parameter r_i through ring j. For a uniform ring thickness Δr, the geometric coefficients $W_{i,j}$ are given by the standard onion-peeling formulation [23, 24]:

$$W_{i,j} = \begin{cases} 0 & (j < i), \\ \dfrac{\Delta r}{2}\sqrt{(2j+i)^2 - 4i^2} & (j = i), \\ \dfrac{\Delta r}{2}\sqrt{(2j+i)^2 - 4i^2} - \dfrac{\Delta r}{2}\sqrt{(2j-i)^2 - 4i^2} & (j > i). \end{cases} \tag{9}$$

We now rewrite the summation equation for the axial stress, Eq. (8), in matrix form as

$$\mathbf{V_1} - \frac{\Delta x}{2\Delta z}\boldsymbol{\beta}_{\mathbf{V_2}} = 2C\,\boldsymbol{\alpha}_{\mathbf{zz}}\,\boldsymbol{\sigma}_{\mathbf{zz}}, \tag{10}$$

where

$$\mathbf{V_1} = \begin{bmatrix} V_1^{(1)} \\ V_1^{(2)} \\ \vdots \\ V_1^{(N-1)} \\ V_1^{(N)} \end{bmatrix}, \quad \boldsymbol{\sigma}_{\mathbf{zz}} = \begin{bmatrix} \sigma_{zz}^{(1)} \\ \sigma_{zz}^{(2)} \\ \vdots \\ \sigma_{zz}^{(N-1)} \\ \sigma_{zz}^{(N)} \end{bmatrix}, \tag{11}$$

and

$$\boldsymbol{\alpha}_{\mathbf{zz}} = \begin{bmatrix} W_{1,1} & W_{1,2} & \dots & W_{1,N-1} & W_{1,N} \\ 0 & W_{2,2} & \dots & W_{2,N-1} & W_{2,N} \\ \vdots & \vdots & \ddots & \vdots & \vdots \\ 0 & 0 & \dots & W_{N-1,N-1} & W_{N-1,N} \\ 0 & 0 & \dots & 0 & W_{N,N} \end{bmatrix}, \tag{12}$$

$$\boldsymbol{\beta}_{\mathbf{V_2}} = \begin{bmatrix} 1 & 1 & \dots & 1 & 1 \\ 0 & 1 & \dots & 1 & 1 \\ \vdots & \vdots & \ddots & \vdots & \vdots \\ 0 & 0 & \dots & 1 & 1 \\ 0 & 0 & \dots & 0 & 1 \end{bmatrix} \begin{bmatrix} V_2'^{(1)} - V_2^{(1)} \\ V_2'^{(2)} - V_2^{(2)} \\ \vdots \\ V_2'^{(N-1)} - V_2^{(N-1)} \\ V_2'^{(N)} - V_2^{(N)} \end{bmatrix}. \tag{13}$$

The axial-stress vector $\sigma_{\mathbf{zz}}$ can then be obtained by multiplying the integrated-value vectors by the inverse of the coefficient matrix, i.e.,

$$\boldsymbol{\sigma}_{\mathbf{zz}} = \frac{1}{2C}\,\boldsymbol{\alpha}_{\mathbf{zz}}^{\mathbf{-1}} \left(\frac{\Delta x}{2\Delta z}\,\boldsymbol{\beta}_{\mathbf{V_2}} - \mathbf{V_1} \right). \tag{14}$$

References

[1] Kavanagh, J.L., Menand, T., Daniels, K.A.: Gelatine as a crustal analogue: Determining elastic properties for modelling magmatic intrusions. Tectonophysics **582**, 101–111 (2013) https://doi.org/10.1016/j.tecto.2012.09.032

[2] Sun, D., Lu, T., Wang, T.: Nonlinear photoelasticity of rubber-like soft materials: Comparison between theory and experiment. Soft Matter **17**(19), 4998–5005 (2021) https://doi.org/10.1039/D1SM00267H

[3] Johnson, K.L.: Contact Mechanics. Cambridge University Press, Cambridge (1985). https://doi.org/10.1017/CBO9781139171731

[4] Yokoyama, Y., Ichihara, S., Tagawa, Y.: High-speed photoelastic tomography for axisymmetric stress fields in a soft material: Temporal evolution of all stress components. Optics and Lasers in Engineering **178**, 108224 (2024) https://doi.org/10.1016/j.optlaseng.2024.108224

[5] Yokoyama, Y., Mitchell, B.R., Nassiri, A., Kinsey, B.L., Korkolis, Y.P., Tagawa, Y.: Integrated photoelasticity in a soft material: Phase retardation, azimuthal angle, and stress-optic coefficient. Optics and Lasers in Engineering **161**, 107335 (2023) https://doi.org/10.1016/j.optlaseng.2022.107335

[6] Mitchell, B., Yokoyama, Y., Nassiri, A., Tagawa, Y., Korkolis, Y.P., Kinsey, B.L.: An investigation of hertzian contact in soft materials using photoelastic tomography. Journal of the Mechanics and Physics of Solids **171**, 105164 (2023) https://doi.org/10.1016/j.jmps.2022.105164

[7] Philippi, J., Lagrée, P.-Y., Antkowiak, A.: Drop impact on a solid surface: Short-time self-similarity. Journal of Fluid Mechanics **795**, 96–135 (2016) https://doi.org/10.1017/jfm.2016.142

[8] Cheng, X., Sun, T.-P., Gordillo, L.: Drop impact dynamics: Impact force and stress distributions. Annual Review of Fluid Mechanics **54**(1), (2022) https://doi.org/10.1146/annurev-fluid-030321-103941

[9] Gordillo, L., Sun, T.-P., Cheng, X.: Dynamics of drop impact on solid surfaces: Evolution of impact force and self-similar spreading. Journal of Fluid Mechanics **840**, 190–214 (2018) https://doi.org/10.1017/jfm.2017.901

[10] Field, J.E.: The physics of liquid impact, shock wave interactions with cavities, and the implications to shock wave lithotripsy. Physics in Medicine & Biology **36**(11), 1475 (1991) https://doi.org/10.1088/0031-9155/36/11/007

[11] Nearing, M.A., Bradford, J.M., Holtz, R.D.: Measurement of force vs. Time relations for waterdrop impact. Soil Science Society of America Journal **50**(6), 1532–1536 (1986) https://doi.org/10.2136/sssaj1986.03615995005000060030x

[12] Soto, D., Larivière, A.B.D., Boutillon, X., Clanet, C., Quéré, D.: The force of impacting rain. Soft Matter **10**(27), 4929–4934 (2014) https://doi.org/10.1039/C4SM00513A

[13] Carré, A., Gastel, J.-C., Shanahan, M.E.R.: Viscoelastic effects in the spreading of liquids. Nature **379**(6564), 432–434 (1996) https://doi.org/10.1038/379432a0

[14] Shanahan, M.E.R., Carre, A.: Viscoelastic Dissipation in Wetting and Adhesion Phenomena. Langmuir **11**(4), 1396–1402 (1995) https://doi.org/10.1021/la00004a055

[15] Xue, N., Wilen, L.A., Style, R.W., Dufresne, E.R.: Droplets sliding on soft solids shed elastocapillary rails. Soft Matter **21**(2), 209–215 (2025) https://doi.org/10.1039/D4SM01041H

[16] Hauer, L., Cai, Z., Skabeev, A., Vollmer, D., Pham, J.T.: Phase Separation in Wetting Ridges of Sliding Drops on Soft and Swollen Surfaces. Physical Review Letters **130**(5), 058205 (2023) https://doi.org/10.1103/PhysRevLett.130.058205

[17] Mangili, S., Antonini, C., Marengo, M., Amirfazli, A.: Understanding the drop impact phenomenon on soft PDMS substrates. Soft Matter **8**(39), 10045–10054 (2012) https://doi.org/10.1039/C2SM26049B

[18] Alizadeh, A., Bahadur, V., Shang, W., Zhu, Y., Buckley, D., Dhinojwala, A., Sohal, M.: Influence of substrate elasticity on droplet impact dynamics. Langmuir **29**(14), 4520–4524 (2013) https://doi.org/10.1021/la304767t

[19] Aben, H.K., Idnurm, S.J., Josepson, J., Kell, K.-J.E., Puro, A.E.: Optical tomography of the stress tensor field. In: Analytical Methods for Optical Tomography, vol. 1843, pp. 220–229. SPIE, ??? (1992). https://doi.org/10.1117/12.131894

[20] Anton, J., Errapart, A., Aben, H., Ainola, L.: A discrete algorithm of integrated photoelasticity for axisymmetric problems. Experimental Mechanics **48**(5), 613–620 (2008) https://doi.org/10.1007/s11340-008-9121-9

[21] Aben, H., Guillemet, C.: Photoelasticity of Glass. Springer, Berlin, Heidelberg (1993). https://doi.org/10.1007/978-3-642-50071-8

[22] Aben, H., Ainola, L., Errapart, A.: Application of the abel inversion in case of a tensor field. Inverse Problems in Science and Engineering **18**(2), 241–249 (2010) https://doi.org/10.1080/17415970903545124

[23] Dasch, C.J.: One-dimensional tomography: a comparison of abel, onion-peeling, and filtered backprojection methods. Applied Optics **31**(8), 1146–1152 (1992) https://doi.org/10.1364/AO.31.001146

[24] Xiong, Y., Kaufmann, T., Noiray, N.: Towards robust BOS measurements for

axisymmetric flows. Experiments in Fluids **61**(8), 178 (2020) https://doi.org/10.1007/s00348-020-03007-4
